# Ex Vivo Expansion of Functional Human UCB-HSCs/HPCs by Coculture with AFT024-*hkirre* Cells

**DOI:** 10.1155/2014/412075

**Published:** 2014-02-25

**Authors:** Muti ur Rehman Khan, Ijaz Ali, Wei Jiao, Yun Wang, Saima Masood, Muhammad Zubair Yousaf, Aqeel Javaid, Shafique Ahmad, Meifu Feng

**Affiliations:** ^1^Department of Pathology, University of Veterinary and Animal Sciences, Lahore 54000, Pakistan; ^2^Institute of Biotechnology and Genetic Engineering, University of Agriculture, Peshawar 25000, Pakistan; ^3^State Key Laboratory of Biomembrane and Membrane Biotechnology, Institute of Zoology, Chinese Academy of Sciences, Beijing 100101, China; ^4^Department of Anatomy and Histology, University of Veterinary and Animal Sciences, Lahore 54000, Pakistan; ^5^Department of Advanced Materials & Nanotechnology, College of Engineering, Peking University, Beijing 100871, China; ^6^Department of Pharmacology and Toxicology, University of Veterinary and Animal Sciences, Lahore 54000, Pakistan; ^7^Institue of Biochemistry and Biotechnology, University of Veterinary and Animal Sciences, Lahore 54000, Pakistan

## Abstract

Kiaa1867 (human Kirre, hKirre) has a critical role in brain development and/or maintenance of the glomerular slit diaphragm in kidneys. Murine homolog of this gene, mKirre expressed in OP9 and AFT024 cells could support hematopoietic stem cells/hematopoietic progenitor cells (HSC/HPC) expansion in vitro. HKirre is also expressed in human FBMOB-hTERT cell line and fetal liver fibroblast-like cells but its function has remained unclear. In this paper, we cloned a hKirre gene from human fetal liver fibroblast-like cells and established a stably overexpressing hKirre-AFT024 cell line. Resultant cells could promote self-renewal and ex vivo expansion of HSCs/HPCs significantly higher than AFT024-control cells transformed with mock plasmid. The Expanded human umbilical cord blood (hUCB) CD34^+^ cells retained the capacity of multipotent differentiation as long as 8 weeks and successfully repopulated the bone marrow of sublethally irradiated NOD/SCID mice, which demonstrated the expansion of long-term primitive transplantable HSCs/HPCs. Importantly, hkirre could upregulate the expressions of Wnt-5A, BMP4, and SDF-1 and downregulate TGF-**β** with other hematopoietic growth factors. By SDS-PAGE and Western Blot analysis, a ~89 kDa protein in total lysate of AFT024-hKirre was identified. Supernatants from AFT024-hkirre could also support CD34^+^CD38^−^ cells expansion. These results demonstrated that the AFT024-hKirre cells have the ability to efficiently expand HSCs/HPCs.

## 1. Introduction

In the past two decades, hematopoietic stem cells from human umbilical cord blood (hUCB-HSCs) have emerged as a promising source of stem cells due to their advantages [1]. However, the low yield of HSCs in hUCB limits its application [2]. HSCs/HPCs expansion has therefore become a much sought-after therapeutic goal of the biomedical sciences as ex vivo expansion may overcome this limitation. However, despite considerable research, ex vivo expansion of hUCB-HSCs has not definitively resulted in improved clinical outcomes, and most of these approaches led to the differentiation and extinction of long-term reconstituting cells (LTRCs) [2, 3].

In stem cell niche, the stromal microenvironment is thought to provide a rich milieu of molecular signals and growth factors that mediate HSCs self-renewal and differentiation. This is achieved through cell to cell adhesion contacts, extracellular matrix deposition, and a combination of cytokines production by the stromal cells [4]. Therefore identification and characterization of these regulators are an active research field [5].

Hkirre is a homolog of Drosophila kirre gene in mammalian having high similarity with Drosophila IrreC-rst and C.elegans SYG-1 [6, 7] an immunoglobulin-like cell adhesion molecule involved in embryonic muscle development and formation of polynucleate myotubes that arise by fusion of myoblasts [8, 9]. MKirre is a homolog of the human hkirre in Murine, which encodes a type Ia membrane protein. The mkirre protein is expressed in founder cells in muscle from mesoderm and contributes to myoblast aggregation in mouse embryonic life [9, 10]. Its extracellular domain has been detected in bone marrow stromal cells [11, 12], heart, and spleen. MKirre gene was isolated from OP9, a mouse bone marrow stromal line, and was demonstrated to play an important role in supporting the HSCs. If expression of mkirre is repressed, OP9 cells significantly lose their ability to support the growth of HSCs. Like other membrane bound growth factors/proteins, the mkirre protein could be cleaved by metalloproteinase while releasing the extracellular domain which is responsible for supporting the HSC [12]. Hkirre has 97% of homologous with mkirre and was also reported to be expressed by AFT024 cells [13]. AFT024 is a stem cell supportive stromal cell line that was derived from murine fetal liver [14], which is an internationally accredited cell line and used as feeder cells.

In our lab, we found that hkirre was also expressed by human fetal bone marrow derived primary stroma and hTERT (define) transfected fetal bone marrow osteoblasts having high hematopoietic supportive capability, which compose the part of bone marrow niche [15]. In the present study, we cloned the Kiaa1867 (human Kirre or hKirre) and established an AFT024 cell line stably overexpressing the hkirre, AFT024-hkirre. We then used these cells to test the effect on ex vivo expansion and maintenance of human UCB enriched CD34^+^ cells in a direct coculture. AFT024-hkirre cells promoted ex vivo expansion and self-renewal of hUCB-HSCs/HPCs significantly higher than control AFT024 cells, a mouse fetal liver cell line. Importantly, the expanded cells retained the multipotency and bone marrow reconstitution ability in vitro and in vivo, indicating that the hkirre is a powerful growth factor to expand multipotent HSCs.

## 2. Materials and Methods

### 2.1. Cell Lines

The mouse fetal liver cell line AFT024 was kindly provided by Moore et al. in Princeton University, Princeton, NJ, which was maintained and subcultured at 33°C as described previously [1, 14]. The cells were irradiated at 1800 (optimized) rads or treated with 10 *μ*g/mL mitomycin for 2 h prior to cocultures.

### 2.2. Cytokines

Human recombinant cytokines were used in the culture systems. Granulocyte-macrophage colony-stimulating factor (GM-CSF) and erythropoietin (EPO) were purchased from R&D Systems (Minneapolis, MN.). Interleukin-3 (IL-3) and IL-6 were purchased from RELIATech (Braunschweig, Germany). Recombinant human stem cell factor (SCF), Flt-3 ligand, and thrombopoietin (TPO) were purchased from Peprotech (London, UK).

### 2.3. Establishment of AFT024 Cells Stably Expressing Hkirre

The 293T packaging cell line (provided by Prof. Guangxia Gao, Institute of Microbiology, Chinese Academy of Sciences, Beijing) was cotransfected with pLPCX-hkirre retroviral plasmid and the viral packaging plasmids (provided by Dr. Haojian Zhang)according toLipofectamine 2000 protocol (Invitrogen, GIBCO, Carlsbad, CA). Viral supernatants were collected at 48 h or 72 h after transfection and used for infecting AFT024 cells. The retroviral supernatant (1.0 mL) containing polybrene (Sigma) at the concentration of 8 *μ*g/mL was added to culture dishes when AFT024 cells grew up to 70%–80% confluent. After incubation for 5 h at 37°C, the transfection medium was replaced by 2.0 mL Dulbecco's modified essential medium (DMEM) purchased from Gibco BRL, Grand Island, NY, supplemented with 10% fetal bovine serum (FBS) (Hyclone Laboratories, Logan, Utah, USA). Puromycin (5 *μ*g/mL) was added to the cultures 24 h later. Puromycin resistant clones grew out from a single cell, and each clone was transferred to a fresh dish for expansion.

### 2.4. Immunoblotting Analysis

AFT024-hkirre cells and control AFT024 cells were cultured for 24 hours and collected in SDS sample buffer and heated for 5 min at 100°C. Immunoblotting was followed as reported by us previously [16]. The proteins were separated by SDS-PAGE and then electrically transferred to polyvinylidene fluoride membranes. Following transfer, the membrane was blocked in TBST (TBS containing 0.1% Tween 20) containing 5% skimmed milk for 2 h, followed by incubation overnight at 4°C with 1 : 500 rabbit anti-flag monoclonal antibody, and 1 : 1000 mouse anti-*β*-actin monoclonal antibody. After washing three times in TBST, 10 min each, the membrane was incubated for 1 h at 37°C with 1 : 1000 horseradish peroxidase-conjugated goat anti-rabbit IgG and horseradish peroxidase conjugated goat anti-mouse IgG, respectively. At final stage, the membrane was processed using the enhanced chemiluminescence detection system (Amersham, Piscataway, NJ).

### 2.5. Isolation of Human Cord Blood CD34^+^ Cells

Human UCB was obtained from normal full-term deliveries with the informed consent of mothers from HaiDian Maternity Hospital, Beijing. UCB was collected into sterile bottles containing anticoagulant citrate-phosphate-dextrose (Sigma, St. Louis, MO, USA). The experiment was approved by the Research Ethics Committees (REC) of the Institute of Zoology (IOZ), Chinese Academy of Sciences (CAS), Beijing, China.

Mononuclear cells were isolated by density gradient centrifuge using lymphocyte separation solution (Ficoll1.077 g/mL from TBD Biotech, Tianjing, China). CD34^+^ cells were enriched by immunomagnetic beads using the MACS CD34, Progenitors Cell Isolation Kit (Miltenyi Biotech Inc., Bergish Gladbach, Germany) following the manufacturer's instructions. The purity of CD34^+^ cells was more than 80% as determined by flow cytometric analysis.

### 2.6. Coculture of hUCB CD34^+^ Cells with AFT024-hkirre Cells

AFT024-hKirre cells or AFT024 control cells transfected with mock plasmid were treated with mitomycin for 2 h (10 *μ*g/mL, optimized after serial dilutions) or ^137^Cs irradiated at a dose of 1800 rads, and 1 × 10^6^ of cells/mL were seeded into 24-well plates coated with 1% gelatin (Nunc A/S, Roskilde, Denmark). Cells were cultured in DMEM and supplemented with 5% FBS (Hyclone Laboratories, Logan, Utah, USA), 10^−4^ 2 mercaptoethanol, 4 mM L-glutamine, 4500 mg/L glucose, 1 mM sodium pyruvate, 1500 mg/L sodium bicarbonate, 5 *μ*g/mL insulin (Sigma Chemical Co., St. Louis, Missouri, USA), 100 U/mL penicillin, and 100 *μ*g/mL streptomycinat 33°C, 5% CO_2_ humidified incubator for 12 to 24 h for proper attachment. HUCB-CD34^+^ cells (1 × 10^5^ cells/mL) were added onto feeder cells treated and transferred to 37°C with 5% CO_2_ incubator. The positive control was grown in stroma free and cytokine cocktail consisting of SCF (10 ng/mL), Flt3 ligand (FL; 10 ng/mL), and thrombopoietin (TPO; 10 ng/mL), and the negative control grown in cytokines and stroma free conditions.

### 2.7. Collection of Supernatants of AFT024-hkirre Cells

To identify the shedding of hkirre protein, the supernatants were collected after culturing 1 × 10^6^ AFT024-hkirre cells per mL for 48 h. The supernatants were centrifuged at 500 g for 30 min at 4°C to remove the cell particles and stored at −20°C for further experiments.

### 2.8. Flow Cytometric Analysis

For the phenotypic analysis of the expanded cells, hematopoietic progenitors cells were harvested by gentle pipetting, counted, and analyzed by flow cytometry after staining with phycoerythrin (PE) conjugated monoclonal antibody to CD34 (PE anti-human CD34 mAb, BD Biosciences) and fluorescein isothiocyanate (FITC) conjugated monoclonal antibody to CD38 (FITC-anti-human CD38 mAb, eBioscience, Inc.) for surface markers expression. The cells were stained for 30 min in the darkness for FITC-conjugated CD38 and PE-conjugated CD34 monoclonal antibodies at 4°C. Samples were blocked with 0.1% BSA (Sigma Chemical Co., St. Louis, Missouri, USA) in PBS before staining to avoid nonspecific binding. FITC or PE-conjugated mouse IgG1 (Becton Dickinson) was used as isotype controls. The stained cells were analyzed using FACsort flow cytometer (Becton Dickinson, San Jose, CA) with CELLQuestTM software (Becton Dickinson).

Additionally, in coculture systems of AFT024 cells and HSCs, the cells attaching to feeder cells were harvested by precise trypsinization and gentle pipetting and then performed flow cytometric analysis to assess the percentage of hematopoietic progenitors.

### 2.9. Long-Term Culture Initiating-Cell (LTC-IC) Assay

For LTC-IC assay, 1 × 10^4^ cells/mL UCB CD34^+^ were plated into six-well plates containing AFT024-hKirre cells or AFT024 control cells treated in LTC medium (Myelo-Cult, StemCell Inc., Vancouver, BC, Canada) along with 10 M hydrocortisone sodium hemisuccinate (Sigma Chemical Co., St. Louis, Missouri, USA). Half of medium was replaced weekly with fresh medium as described previously [17]. Adherent and nonadherent cells were harvested weekly during 5–8 weeks of coculture and transferred to 1.8% methylcellulose medium (it was dissolved in distilled water at the concentration of 1.8%, autoclaved the solution, and then put it at 4°C for gradual and complete salvation) containing 2.8% BSA, 30% FBS (Hyclone), 50 ng/mL SCF, 20 ng/mL IL-3, 20 ng/mL GM-CSF, 20 ng/mL IL-6, and 3 U/mL EPO at 37°C with 5% CO_2_ in humidified incubator. After 14–16 d of cultures, colonies with greater than 50 cells were counted to assess LTC-IC activities. Colony-forming units (CFU-Cs) and lineage-specific colonies including colony-forming unit-erythrocyte (BFU-Es), colony-forming unit granulocyte-macrophages (CFU-GMs), and colony-forming unit mix (CFU-Mix's) were counted using an inverted microscope (Nikon; Sesto Fiorentino, Italy) and calculated the frequency of LTC-IC according to the manufacturer's instructions (StemCell Inc.).

### 2.10. NOD/SCID Repopulating Cell (SRC) Assay

NOD/SCID mice were purchased from Animal Center of Chinese Medical Academy of Sciences and kept in the animal facilities of the Institute of Zoology, Chinese Academy of Sciences, Beijing, China. Animals were housed under specific pathogen-free conditions in filter-top cages provided with sterilized food and drinking water ad libitum. During transplantation procedure, they were handled in sterile cabinets. All experiments were performed according to a study protocol approved by the REC of IOZ, CAS. After coculturing with hkirre-AFT024 cells or AFT024 control cells, 5 × 10^3 expanded cells suspended in 1 mL *α*-MEM were injected intravenously into 8-week-old, sublethally irradiated (3.5 Gy) NOD/SCID mice. Positive or negative control mice were transplanted with freshly isolated CD34^+^ cells or *α*-MEM, respectively. The mice were sacrificed at 8 wks after transplantation; bone marrow MNCs were harvested by Ficoll density gradient centrifuge and stained with FITC-labeled anti-human CD45^+^ antibody and then analyzed by flow cytometry. For confirmation of flow cytometry results, human specific 17  *α*-satellite gene expression was checked by PCR analysis after isolating DNA from MNCs harvested from mice bone marrow. DNA was denatured at 95°C for 5 minutes and then cooled down to 54°C for primer annealing. Amplification was done until 32 cycles at 72°C for 35 seconds. The cut-off level of SRCs was determined by the results of flow cytometry and PCR.

### 2.11. RT-PCR and Semiquantitative PCR Analysis

The TRIzol Reagent (Life Technologies Inc. Gaithersburg, MD) was use to extract total RNA from the different groups of cultured cells. Genomic DNA contamination was removed by DNAse1. The mRNA was reverse-transcribed into the first-strand cDNA using oligo (dT) primer. The RT reaction was performed for 1 h at 42°C and stopped by heat inactivation for 10 min at 70°C. The cDNA was subjected to PCR amplification with specific primers under linear conditions. As the above step in PCR amplification, cDNA was denatured at 95°C for 5 minutes following the annealing temperatures of primers and elongation at 72°C for 35 seconds. The primers used in the study are listed in Table 2 (supplemental data). Following the amplification, each reaction mixture was visualized by 1.0% agarose gel electrophoresis.  *β*-Actin was served as an internal control.

For semiquantitative RT-PCR, the amount of each mRNA was assessed up to 29 to 35 cycles of PCR. Densitometric analysis was performed using Fluor-STM Multi-imager and Quality One 4.3.0 software (Bio-RAD, Hercules, CA).

### 2.12. Statistical Analysis

The data were represented as mean ± SD. Statistical comparisons were performed using a two-tailed Student's *t*-test. *P* values less than 0.05 were recognized as significant.

## 3. Results

### 3.1. Hkirre Supports Ex Vivo Expansion of Hematopoietic Progenitor Cells

**Figure 1:**
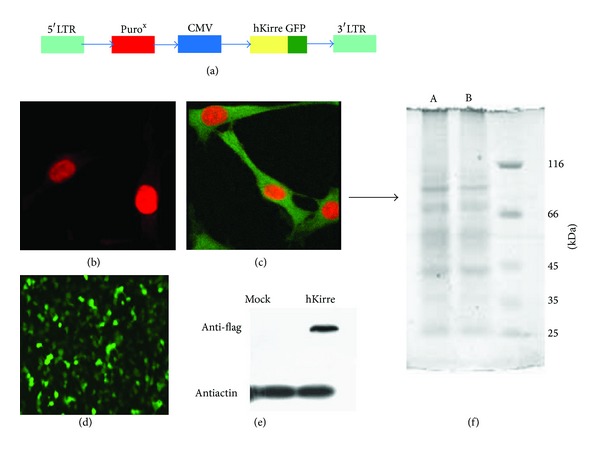
Reconstruction of AFT024-hKirre cells and expression of hKirre-GFP protein. (a) Recombinant hkirre-GFP retrovirus vector design: LTR indicates long terminal repeat; puro, puromycin-resistant gene; PCMV IE, cytomegalovirus (CMV) immediate early promoter; hKirre-GFP, human Kirre attached with GFP protein. (b) AFT024 cells transfected with mock plasmid (×400). (c) AFT024 cells transfected with hkirre plasmid (×400). (d) AFT024-hkirre cells (×100). Images (b) to (d) showed a high transfection efficiency under fluorescence microscope. (e) Western Blot analysis of hkirre expression in the AFT024 transformed cells and control cells. (f) Total AFT024-hkirre cells lysate was checked for the hkirre protein on SDS page and stained with commassive brilliant blue (stain). A differentially expressed protein of approximately 89 kDa was detected (A) compared with the control cells (B).

The results about reconstruction of AFTO24 cell line and expression of hkirre-GFP protein were showed in Figure 1.

The hUCB enriched CD34^+^ cells were cocultured with AFT024-hkirre cells or AFT024 control cells after irradiation and cultured with cytokines conditioned medium or medium (DMEM) alone. After the cells were cocultured for seven days, the hUCB CD34^+^ cells were counted and analyzed by flow cytometry for CD34^+^ and CD38^−^ surface markers. The AFT024-hkirre cells significantly increased the total number of CD34^+^ cells as well as CD34^+^ CD38^−^ cells, as compared to AFT024 control cells. Both CD34^+^ CD38^−^ and CD34^+^ CD38^+^ cells increased ~60.47 ± 20.52 and 45.15 ± 21.45 folds, respectively, from the initial cell numbers (Table 1). Importantly, the number of CD34^+^CD38^−^ cells increased, which represented the primitive hematopoietic cells. These results reveal that hKirre enhances the ability of AFT024 cells to promote the expansion and self-renewal of primitive hematopoietic progenitor cells.

Table 2 was the list of primers used in this study as a supplemental data in the end of paper.

### 3.2. Hkirre Protein Supports the Expansion of Primitive HSCs/HPCs

**Figure 2:**
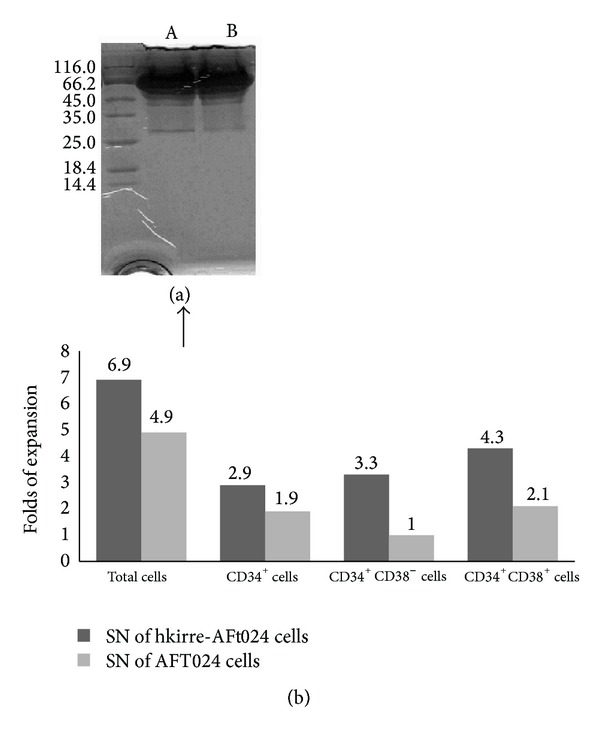
Shedding of hkirre extracellular motif in supernatants and its supportive activity. (a) 1 × 10^6^/mL AFT024-hkirre cells, (A) and control cells (B) were cultured for 48 hours and supernatants were collected. A protein of roughly 27 kDa was detected by SDS-PAGE stained with commassive brilliant blue. (b) 1 × 10^5^ CD34^+^ cells/well in 24-well culture plates were cultured with supernatants of AFT024-hKirre cells or AFT024 control cells. All cells were harvested from wells by gentle pipetting and counted with hemocytometer. For flow cytometry analysis, cells were stained with PE-conjugated mAb to CD34 and FITC-conjugated mAb to CD38. Values indicate the fold increase compared with the initial number of cells. The results were expressed as mean ± SD (*n* = 4). *P < 0.05 and **P < 0.01 compared between SN of AFT024-hkirre and AFT024 control cells (Student's *t*-test).

To investigate the activity and shedding of hkirre protein outer motif in the supernatants (SN), we cultured AFT024-hkirre cells (1 × 10^6^ cells/mL) and collected the supernatants after 48 hours. Over 7 days of culture with SN, the expansion of hUCB-HSCs/HPCs promoted, especially the CD34^+^CD38^−^ cells fraction (3.33 ± 1.11), was significantly higher than control (1.05 ± 0.37) by flow cytometry analysis. From these results, we predicted the shedding of extracellular portion of hkirre protein and its supportive role and detected a protein of roughly 27 kDa by SDS-PAGE stained with commassive brilliant blue (Figure 2).

### 3.3. AFT024-hkirre Cells Promote LTC-IC of HSCs/HPCs

**Figure 3:**
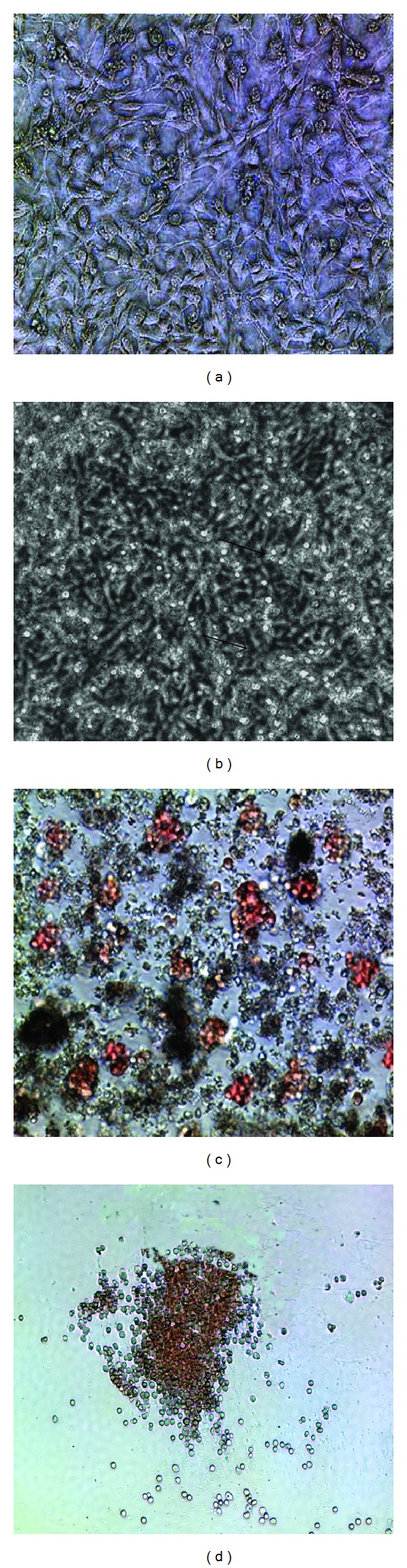
hUCB-CD34^+^ cells cocultured with AFT024-hkirre and CFU assay. (a) AFT024-hkirre feeder cells at 100% confluence. (b) Picture of AFT024-hkirr cells cocultured with CD34^+^ cells. Picture was made on the 7th day of coculture. The bright contrast cells are attached or floating, and dark contract cells are underneath migrated HSCs. (c) The picture of CFU-Mix, showing the granulocytic and erythrocytic colonies. (d) The picture of CFU-GM on day 16. All pictures were taken at 100x.

**Figure 4:**
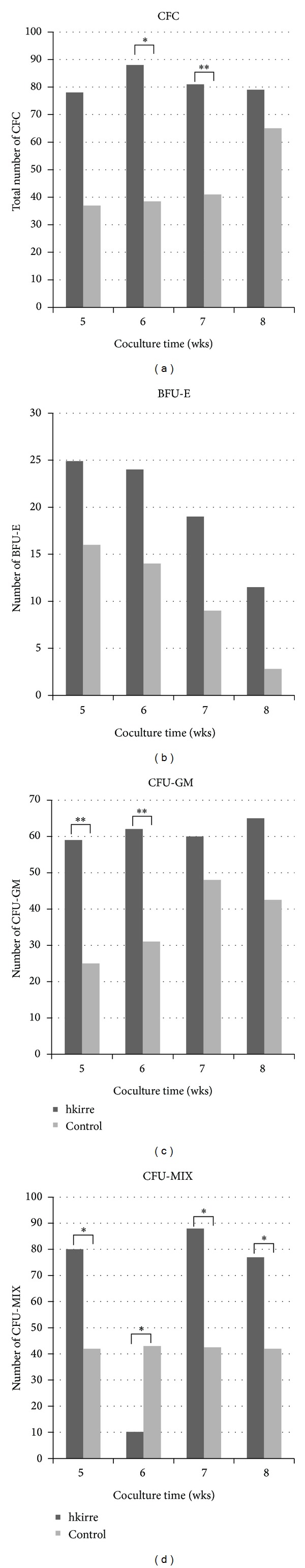
The effect of the AFT024-hKirre cells on LTC-IC assay of CD34^+^ cells. 1.0 × 10^4^ CD34^+^ CB cells were cocultured with AFT024-hkirre cells or AFT024 control cells for 5–8 weeks and then subjected to CFU assay. After 16 days of culture, the colonies, including BFU-Es, CFU-GMs, and CFU-Mix's, with greater than 50 cells were counted. The results are expressed as mean ± SD (*n* = 3). *P < 0.05 and **P < 0.01 compared between AFT024-hkirre and AFT024 control cells (Student's *t*-test).

To further confirm the capability of AFT024-hkirre cells in supporting the self-renewal and maintaining the multipotential differentiation of HSCs/HPCs, LTC-IC assay was performed. HUCB-CD34^+^ cells were cocultured with AFT024-hkirre or control cells treated in LTC-IC medium for 5–8 weeks and then subjected to colony forming units assay. After 16 days of culture, the colonies with greater than 50 cells were counted. As shown in Figure 3, the number of total Colony Forming Cells (CFCs) from the hUCB CD34^+^ cells cocultured with AFT024-hkirre cells for 6-7 wks was significantly higher than that cocultured with control AFT024 cells. Consistently, the number of BFU-Es, CFU-GMs, and CFU-Mix's was also higher in the case of cocultures with AFT024-hkirre cells than the AFT024 control cells. The results suggest that the hkirre could increase AFT024 cells capability of expansion and maintenance of multipotent hematopoietic progenitor cells as hUCB CD34^+^ cells retained the capacity of multipotent differentiation at least for 8 weeks and the number of CFU-Mix's and CFU-GMs was nonreduced at this time compared with 5 wks of culture. The CD34^+^ cells cocultured with AFT024-hkirre have superior LTC-IC activity (Figure 4).

### 3.4. Expanded Cells Successfully Reconstitute the Bone Marrow of NOD/SCID Mouse (SRC Assay)

**Figure 5:**
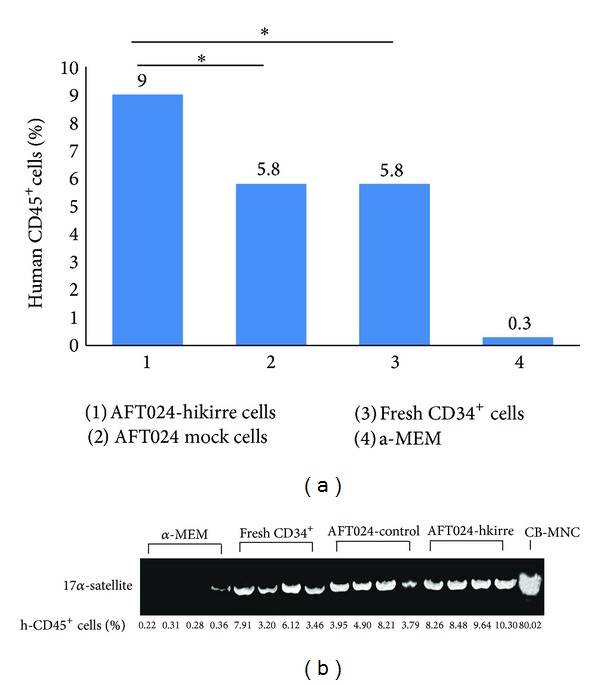
Effect of AFT024-hkiree cells on the reconstitution ability of CD34^+^ cells in NOD/SCID mice. UCB-HSCs were coculture with AFT024-hkirre or control cells and harvested after 4 week. 3 × 10^3^ cells were injected intravenously into sublethal irradiated NOD/SCID mice. For positive control, freshly isolated CD34^+^ cells, and for negative control, only medium was injected. Each group comprises of three mice at least. After 8 weeks of transplantation, mice were sacrificed,and mononuclear cells were harvested and analyzed for human CD45^+^ cells by flow cytometry ((a) graph showing the percentage of hCD45^+^ cells and (b) for the expression of human specific 17  *α*-satellite gene by PCR). The results are expressed as mean ± SD (*n* = 4). *P < 0.05 and **P < 0.01 compared between AFT024-hkirre and AFT024 and AFT024-hkirre and freshly isolated CD34^+^ cells (Student's *t*-test).

To determine the bone marrow repopulation capability of ex vivo expanded hUCB HSCs/HPCs, we performed the transplantation experiments to know the engraftment of SRCs in NOD/SCID mice. The mice radiated sublethally (3.5 Gy) were transplanted with 3 × 10^3^ CD34^+^ cells cocultured with AFT024-hkirre cells or control cells treated. As positive control, freshly isolated CD34^+^ cells were transplanted, and as negative control we only injected medium. Mice were sacrificed after 8 weeks after transplantation and analyzed for human CD45^+^ cells percentage in mice bone marrow by flow cytometry. The results were expressed as mean ± SD (*n* = 4). *P < 0.05 and **P < 0.01, compared between AFT024-hkirre and AFT024, and AFT024-hkirre and freshly isolated CD34^+^ cells (AFT024-hkirre 9.17 ± 0.97, AFT024 5.21 ± 2.06, freshly isolated CD34^+^  5.17 ± 2.25, *P < 0.05), (Figure 5(a)). The human specific 17 alpha satellite gene was also checked by PCR when CD45 ^+^ cells percentage was higher than 0.36%. The cut-off level of engraftment was determined at 0.40% of hCD45^+^ cells (Figure 5(b)). These results showed that AFT024-hkirre cells promoted the expansion of long-term primitive transplantable HSCs.

### 3.5. Expression of Hematopoiesis-Related Factors in Coculture System of AFT024-hkirre Cells with CD34^+^ Cells

**Figure 6:**
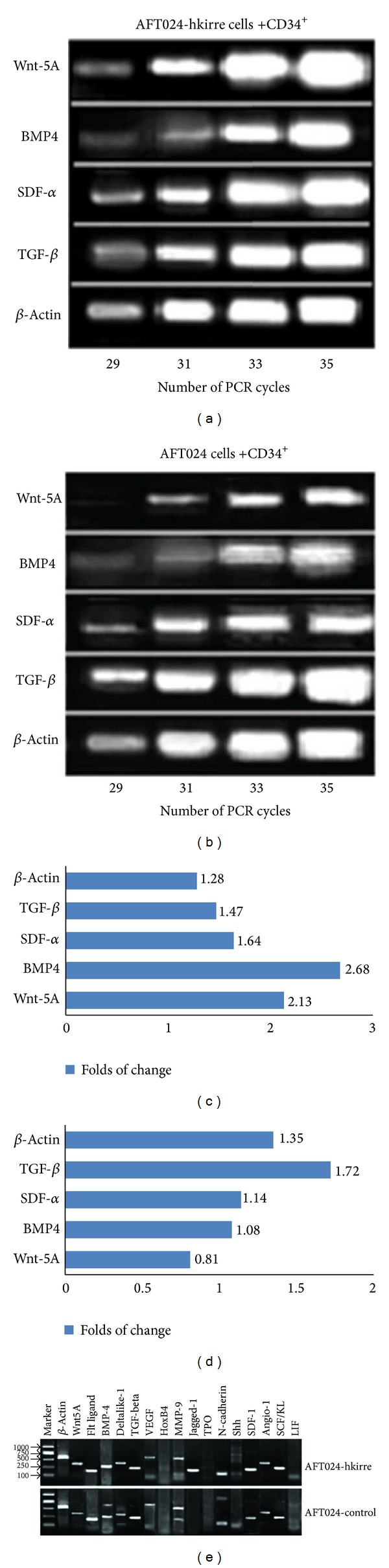
Genes expression profile of AFT024 cells interacted with CD34^+^ cells and semiquantitative RT-PCR of selected genes. The figure shows the effect of interaction between hUCB-HSCs/HPCs and AFT024-hkirre cells on the transcription of Wnt-5A, BMP4, SDF-*α*, and TGF-*β*. The AFT024-hkire cells (1.0 × 10^6^) were cocultured with CD34^+^ cells (1.0 × 10^5^/well) in six-well plates for 24 hr and then washed twice to remove loosely adherent and nonadherent cells. The adherent cells were harvested and analyzed by semiquantitative RT-PCR for transcripts of the above-mentioned genes. Fold changes at 35 cycles were determined through densitometric analysis using Fluor-STM Multi-imager and Quality One 4.3.0 software (Bio-RAD) (a) expression from AFT024-hkirre cells, (b) expression from the AFT024-control cells, (c) folds of change from AFT024-hkirre group, and (d) folds of changes from control group. The data shown was a representative experiment of three reproducible experiments. (e) Cytokine (profile) expression profile was analyzed by RT-PCR. Upper panels show AFT024-hKirre cells group, and lower panels show the AFT024 control cells group.

After successful expansion and evaluation of HSCs/HPCs expanded in coculture with AFT024-hkirre, the gene expression of hematopoietic-related factors in this system was assayed. AFT024-hkirre cells and control cells were cocultured with CD34^+^ cells for 24 h. AFT024-hkirre and control cells were lysed by TRIzol after washing off loosely adherent and nonadherent CD34^+^ cells. Total mRNA was isolated and analyzed by RT-PCR. The results showed that Wnt-5A and BMP-4 transcription factor mRNAs were expressed but HoxB-4 mRNA was not detected. SDF-1, TGF- *β*, SCF/KL, FL, Dlk-1, Angio-1, MMP-9, and Jagged-1 mRNAs were expressed but not TPO, Shh, and LIF (Figure 6(e)). These results suggest that hkirre transformation promoted the expression of hematopoietic related factors in the coculture system, which provided a rich milieu of molecular signals that mediate HSC self-renewal and expansion by creating a suitable niche environment.

### 3.6. Hkirre Gave Support to Hematopoiesis through SDF-1/TGF *β*-Induced Control of the Cycling/Quiescence and Transcriptional Upregulation of Wnt5A and BMP-4

On the basis of RT-PCR results mentioned above, we decided to quantify the expression of Wnt5A, BMP-4, SDF-1, and TGF-*β* to explore the molecular mechanism by which hkirre enhanced the ability of AFT024 cells in maintaining and promoting ex vivo expansion of HSCs/HPCs. Five regulatory genes were analyzed by semiquantitative RT-PCR. Reactions were carried out for an increasing number of cycle steps (two-cycle increments) for all genes. Total mRNA was isolated from the AFT024-hKirre and AFT024 control cells after 24 h of coculture with CD34^+^ cells. Expression levels were normalized using *β*-actin as internal control, and the fold changes were determined by densitometric analysis. Levels of Wnt5A (2.13-fold change), BMP-4 (2.68-fold change), and SDF-1 (1.64-fold change) exhibited upregulated expression in AFT024-hkirre, except TGF-*β* expression was higher in control group (1.72-folds). These results demonstrated that the transcripts of Wnt-5A, BPM-4, and SDF-1 were significantly increased in the AFT024-hkirre cells cocultured with UCB CD34^+^ cells. In contrast, the transcripts of inhibitory cytokine TGF-*β* decreased in the AFT024-hkirre cells upon interaction with CD34^+^ cells (Figures 6(a)–6(d)).

## 4. Discussion

Balance between self-renewal and differentiation is determined by the microenvironment which consists of stem cells, their progeny, osteoblasts, stromal cells, and adipocytes, named as niche [18]. How niches modulate self-renewal remained a challenge for scientists [19]. The self-renewal and differentiation of HSCs rely on the specified microenvironments, which is poorly defined in mammals. Searches are going on for hematopoietic-related genes from the niches like bone marrow in adult and aorta gonado mesonephric (AGM) region from developing embryo. Previous studies have demonstrated that the hkirre is expressed in bone marrow stromal cells and also osteoblasts cells [15], but its role remained undefined. Here we have demonstrated its role in expansion and self-renewal of HSCs/HPCs.

We cloned the hkirre (kiaa1867) and reconstructed the AFT024 cell line. The hkirre was stably overexpressed in AFT024 cell line and a protein of approximately 89 kDa was identified in total lysate of cells. AFT024-hkirre cells supported HSCs/HPCs expansion significantly higher than control cells in the coculture. Phenotypic analysis of expanded cells by flowcytometry revealed the higher proportion of CD34^+^CD38^−^ cells representing long-term repopulating cells (LTRC) than CD34^+^CD38^+^ cells which exhibit only for short-term repopulating cells (STRC) [20–22]. Our results are consistent with previous findings that UCB LTC-IC were present among the CD34^+^ cell fraction [23] and LTC-IC assays exhibit multilineage differentiation ability and major proliferative potential, which was regarded as a functional measure of self-renewal [24]. Furthermore, we performed SRC assay which is the best reliable research tool for in vivo analysis of human hematopoietic [25]. The SRC assay indicated the reconstituting ability of these cultured cells. Difference in the percentage of chimerism of human CD45^+^ cells between bone marrow cells of mice transplanted with cultured cells and those transplanted with control samples strongly suggested the extensive ability of these ex vivo-generated HSCs to sustain and reconstitute long-term human hematopoiesis in vivo. PCR analysis also confirmed flow cytometric results [20]. The expanded hematopoietic progenitors are, therefore, capable to sustain long-term hematopoiesis.

The expansion and maintenance of HSCs/HPCs are probably associated with the enhanced expression of BMP-4 and Wnt5A, two important transcription factors for HSCs expansion, and maintenance. Wnt5A signaling not only provides proliferative stimuli but also regulates HSCs fate during hematopoietic [26] and augments repopulating capacity and primitive hematopoietic development of human blood stem cells [27]. Similarly BMP4 can promote both proliferation and survival signals [28–30] affecting the cycling or self-renewal capacity of primitive hematopoietic cells.

Secondly, the expression of SDF-1 and TGF-*β* may be another possible mechanism. The hkirre promoted the expression of SDF-1 and comparatively downregulated TGF-*β* genes in our system which may create a balance in cell cycle regulation as SDF-1 modulated the expression of key cell cycle regulators such as cyclins, cyclin dependent kinase inhibitors, and TGF-*β* target genes, confirming its cell cycling promoting effect, and counter acts on the inhibitory effect of TGF-*β* [31]. SDF-1/TGF-*β* induced control of the cycling/quiescence switch via FoxO3a and mTOR pathways. They act as connectors and propose a model integrating a dialogue between the two molecules in cell cycle progression [32]. Moreover, SDF-1 and its receptor CXCR4 play a pivotal role in the homing and repopulation of CD34^+^ SRC in NOD/SCID mice [33]. Hkirre promoted the upregulation of Dlk-1 which already have been reported to be expressed in AFT024 cells and contribute to the HSC expansion and maintenance [34, 35]. Similarly FL [36], SCF [37], N-cadherin [38], and VEGF [39] expression in our culture system provided strong signals for self-renewal and expansion.

We also demonstrated that the SN of AFT024-hkirre cells coculturing with HSCs/HPCs had a greater activity of hematopoietic stem cell expansion as compared to AFT024 control cells. A protein of ~27 kDa was detected in SN from AFT024-hkirre cells and a similar protein in lower concentrations was found in control cells, confirming the expression of mkirre in AFT024 cells as reported previously [13]. Hkirre protein is cleaved by proteolytic mechanism as observed in other membrane bound growth factors, receptors, or adhesion molecules, which is quite similar to SCF regulated by MMP9 [40]. These findings are similar to shedding of mkirre and neph2 [12, 41], and hkirre protein was reported similar to Neph2 expressed in kidney podocytes [9]. NEPH2 is highly expressed in the mammalian muscle and brain. It has been suggested that NEPH2 controls synapse and myocyte formation during mammalian development [41]. More information about the function of this gene is still not available. Homolog of NEPH2 gene in mice has been reported to support hematopoietic stem cells ex vivo culture, and comparison of mKirre and Kiaa1867 proteins was done by Ueno et al. in 2003 [12]. Here we have reported for the first time the hkirre role in expansion and maintenance of hUCB-HSCs/HPCs although the mechanism underlying this function is largely unclear and needs further investigations. We have further hypothesized to identify its receptor on the human HSCs and to generate corresponding knockout mice for further understanding of its function and mechanism by which hematopoiesis is regulated. The outer motif of hkirre protein is under trials to use it as a new cytokine in further hUCB HSC/HPCs expansion.

In conclusion, the main obstacle to hUCB transplantation in adult recipients is the insufficiency of hematopoietic progenitors. In this paper, we tried to seek expansion of tissue specific stem cells while retaining their multipotency and undifferentiation in vitro. A novel AFT024-hkirre coculture system was successfully established. We demonstrated that hkirre significantly supported HSC self-renewal and expansion of multipotent. Its biological activity in SN raised the possibility to use it as a new cytokine for HSC expansion strategies.

## Figures and Tables

**Table 1:** Umbilical cord blood HSCs expansion over seven days of culture.

Groups	AFT024-hKirre cells	AFT024 control cells	Stroma free (cytokines added)	Stroma free (without cytokines)
CD34^+^ cells	14.87 ± 2.54*	7.88 ± 0.74*	15.54 ± 5.35	2.86 ± 1.19
CD34^+^CD38^−^ cells	60.47 ± 20.52*	24.32 ± 8.83*	14.91 ± 2.73	0.93 ± 0.23
CD34^+^CD38^+^ cells	45.15 ± 21.28	41.73 ± 10.58	58.60 ± 13.46	8.84 ± 5.34

Cells were analyzed by flow cytometry after staining with PE-conjugated mAb CD34 and FITC-conjugated mAb CD38. Values indicate fold increase based on the initial number of cells (1 × 10^5^). The results are expressed as mean ± SD (*n* = 5). *P < 0.05 versus control cells (two-tailed Student's *t*-test).

**Table 2:** List of primers used in this study.

Gene	Primers	Product size (bp)
TPO	5′-TTC CAA CAC CTG CTC CG-3′	334
	5′-CCT TGT GGA GTG CGT GAA-3′	

SCF	5′-CAG GAA AGA GTC CAC GAG TT-3′	323
	5′-TGG CTG CCC AGT GTA GG-3′	

Beta-Actin	5′-AGCGGGAAATCATGCGT-3′	515
	5′-CTA GAA GCA TTT GCG GTG G-3′	

FLt3	5-CTGGAGCCCAACAACCTATC-3′	181
	5′-GGA CGG TGA CTG GGT AAT CT-3′	

MMP9	5′-TCCCTGGAGACCTGAGAACC-3′	519
	5′-AATGGGCGTCTCCCTGAAT-3′	

Jagged-1	5-GAT CCT GTC CAT GCA GAA CG-3	440
	5-GGA TCT GAT ACT CAA AGT GG-3	

Wnt-5A	5-ACA CCT CTT TCC AAA CAG GCC-3	341
	5-GGA ATT GTT AAA CTC AAC TCT C-3	

HOXB4	5′-TCA CAG AGC GAT TAC CTA CCC A-3′	498
	5′-CCG TGT CAG GTA GCG GTT GT-3′	

Delta-like 1	5′-AGA CGG AGA CCA TGA ACA ACC-3′	341
	5′-TGA AGT TGA ACA GCC CGA GT-3′	

Angio 1	5′-AAT AAT ATG CCA GAA CCC-3′	500
	5′-AGA TAG TCA ACC TAC GAA A-3′	

TGF-b	5′-CAA GTG GAC ATC AAC GGG TT-3	297
	5′-GCT CCA AAT GTA GGG GCA GG-3′	

SDF alpha	5′-CAC TCC AAA CTG TGC CCT TCA-3′	200
	5′-TCC TTT TCT GGG CAG CCT T-3′	

Shh	5′-ACT GGG TGT ACT ACG AGT CCA AGG-3′	211
	5′-AAA GTG AGG AAG TCG CTG TAG AGC-3′	

17 *α*-satellite	5′-ACG GGA TAA CTG CAC CTA AC-3′	234
	5′-CCA TAG GAG GGT TCA ACT CT-3′	

LIF	5′-GCC AAC GGC ACG GAG AA-3′	128
	5′-TTG CTG TGG AGG CTG AGG G-3	

BMP-4	5′-CAGCAGCATCCCTGAGAACGAG-3′	306
	5′-GTCCGAGTCTGATGGAGGTGAG-3′	

VEGF	5′-TCG GGC CTC CGA AAC CAT GA-3′	649
	5′-CCT GGT GAG AGA TCT GGT TC-3′	
